# The relationship between job satisfaction, burnout, and turnover intention among physicians from urban state-owned medical institutions in Hubei, China: a cross-sectional study

**DOI:** 10.1186/1472-6963-11-235

**Published:** 2011-09-24

**Authors:** Yimin Zhang, Xueshan Feng

**Affiliations:** 1 Department of Social Medicine, School of Public Health, Fudan University, 130 Dongan Road, Shanghai 200032, China; 2 Pudong Institute For Health Development, 818 Laiyang Road, Shanghai 200129, China; 3 Key Laboratory of Public Health Security, Ministry of Education, 130 Dongan Road, Shanghai 200032, China

## Abstract

**Background:**

Throughout China, a growing number of physicians are leaving or intending to depart from their organizations owing to job dissatisfaction. Little information is available about the role of occupational burnout in this association. We set out to analyze the relationship between job satisfaction, burnout, and turnover intention, and further to determine whether occupational burnout can serve as a mediator among Chinese physicians from urban state-owned medical institutions.

**Methods:**

A cross-sectional survey was carried out in March 2010 in Hubei Province, central China. The questionnaires assessed sociodemographic characteristics, job satisfaction, burnout, and turnover intention. The job satisfaction and occupational burnout instruments were obtained by modifying the Chinese Physicians' Job Satisfaction Questionnaire (CPJSQ) and the Chinese Maslach Burnout Inventory (CMBI), respectively. Such statistical methods as one-way ANOVA, Pearson correlation, GLM-univariate and structural equation modeling were used.

**Results:**

Of the 1600 physicians surveyed, 1451 provided valid responses. The respondents had medium scores (3.18 +/-0.73) on turnover intention, in which there was significant difference among the groups from three urban areas with different development levels. Turnover intention, which significantly and negatively related to all job-satisfaction subscales, positively related to each subscale of burnout syndrome. Work environment satisfaction (*b *= -0.074, *p < 0.01*), job rewards satisfaction (*b *= -0.073, *p < 0.01*), organizational management satisfaction (*b *= -0.146, *p < 0.01*), and emotional exhaustion (*b *= 0.135, *p < 0.01*) were identified as significant direct predictors of the turnover intention of physicians, with 41.2% of the variance explained unitedly, under the control of sociodemographic variables, among which gender, age, and years of service were always significant. However, job-itself satisfaction no longer became significant, with the estimated parameter on job rewards satisfaction smaller after burnout syndrome variables were included. As congregated latent concepts, job satisfaction had both significant direct effects (gamma_21 _= -0.32, *p < 0.01*) and indirect effects (gamma_11 _× beta_21 _= -0.13, *p < 0.01*) through occupational burnout (62% explained) as a mediator on turnover intention (47% explained).

**Conclusions:**

Our study reveals that several, but not all dimensions of both job satisfaction and burnout syndrome are relevant factors affecting physicians' turnover intention, and there may be partial mediation effects of occupational burnout, mainly through emotional exhaustion, within the impact of job satisfaction on turnover intention. This suggests that enhancements in job satisfaction can be expected to reduce physicians' intentions to quit by the intermediary role of burnout as well as the direct path. It is hoped that these findings will offer some clues for health-sector managers to keep their physician resource motivated and stable.

## Background

Accompanied by an improvement in the social security system and reform of labor and employment mechanisms in China, the phenomenon of voluntary turnover has extended gradually from corporations to such sectors as public institutions [1]. Some recent studies have brought to light that a growing number of clinical physicians in public hospitals are leaving or intending to depart from their organizations [2,3]. Further, an investigation (n = 3182) by the Chinese Medical Doctor Association in 2009 indicated that up to 44.82% of physicians wanted to give up the medical profession [4]. Though turnover is to some extent favorable for the optimal allocation of human resources, high turnover rates clearly affect the sustainable development of organizations and also deeply disrupt the morale of the employees that remain [5]. Especially in the case of physicians, who require a very long education and training period, high turnover rates can result in enormous transition costs and the loss of patient confidence, which are extremely serious problems for hospitals. Thus, in this context, ensuring the stability of medical teams in state-owned medical institutions, which have been recognized as the most important and also most difficult area in a new round of medical system reform throughout China, has become an urgent issue that needs to be addressed.

The earliest study on turnover was the participant determination model constructed by March & Simon in 1958 [6]. Then Mobley et a1 proposed a range of typical theories between 1977 and 1979, including the decision-making process model, the employee-withdrawal behavior model, and intermediate chain extension model [7-9]. Afterwards, Bluedorn (1982), Sheridan & Abelson (1983), and Lee & Mitchell (1994) further advanced the integration model, the peak mutation model, and the unfolding model, respectively [10-12]. But the most considerable influence so far has been the loss motivation model by Price & Mueller (1977, 2000) [13,14]. On the one hand, these theoretical models and studies indicated that turnover intention was the principal cognitive precursor of turnover behavior with greatly explanatory power [15,16]. On the other hand, turnover intention could also better reflect the real level of organization management compared with turnover behavior, which is easily affected by external factors. Based on the two aspects, it is considered more meaningful to discuss turnover intention rather than actual turnover behavior. Thus, turnover intention, not behavior, is the theme of the present study.

It can be seen from some of the above advocated models that job satisfaction has been deemed the most representative antecedent variable to directly anticipate the turnover intention, and this is manifest in many empirical studies [1,17,18]. In contrast to this view, the integration model, the loss motivation model, and other studies support the view that job satisfaction may exert an impact on turnover intention mainly through organizational commitment [10,14,19-21]. Moreover, a third opinion has demonstrated that both direct and indirect paths could exist at the same time [22,23]. However, these studies on the relationship between job satisfaction and turnover intention have mostly focused on corporate employees; data is still rare among the population of physicians, though several surveys have been attempted in different countries [24-26]. In the case of China, to motivate and keep physicians from quitting urban state-owned medical institutions, further studies are necessary.

Burnout is a comprehensive concept, first proposed by Freudenburger in clinical psychology (1974) [27]. According to the famous structure defined by Maslach C [28], burnout stemming from continuous work pressures that are not effectively handled comprises the following three dimensions: emotional exhaustion, depersonalization, and reduced personal accomplishment. The correlation between job satisfaction and burnout has been confirmed in health care, and more recent studies have noted that a few dimensions of job satisfaction can significantly contribute to occupational burnout among physicians [29,30], though controversy exists in this area. Furthermore, rich evidences have suggested that turnover intention is more likely to occur for employees with high-degree burnout in many professions [31,32]. In spite of many efforts to probe the relationship between pairs of these factors, no published paper has concerned itself with the integral interrelationship of all three concepts of job satisfaction, burnout, and turnover intention. Thus, there is little information about the role of burnout in the association between job satisfaction and turnover intention.

From the above summary and the attitude theory in psychology revealing connections among three attitude components, our primary hypothesis in this study was as follows: there may be an intermediary role of occupational burnout, placing additional emphasis on the emotional element of job attitude in the relationship between job satisfaction (concentrating on the cognitive and appraisal element of job attitude) and turnover intention (concentrating on the behavior intention element of job attitude). The aim of this paper is to describe the degree of turnover intention and compare differences among groups; to estimate actual effect of job satisfaction and burnout syndrome dimensions on turnover intention, based on the correlation examined; and to further verify assumed model on the synthesized relationship among job satisfaction, occupational burnout, and turnover intention as congregated latent concepts to determine whether burnout could serve as a mediator among Chinese physicians from urban state-owned medical institutions.

## Methods

### Participants and sampling

Hubei province in central China, where the number of doctors per thousand population is average, was determined as the source site of sample. Consequently, all physicians in urban state-owned medical institutions of this province who had a practicing qualified certificate on file were eligible for admittance to this study. Based on available official data, the total number of physicians in the study population was 12568, accounting for 45% of the total of physicians from this province. From the data of an initial investigation (n = 276) undertaken in one city, Hubei province, the sample size in actual survey was calculated to be no fewer than 1445 individuals for estimating population mean of the turnover intention of physicians by the following determination formulas: n = t^2^_α/2_σ^2^/δ^2^, Z _a/2 _instead of t_a/2_, a = 0.05, δ = 0.05, σ = 0.75; n_1 _= n × deff (design effect, 1.5); n_C _= n_1_/(1-n_1_/N), N = 12568. A method of multistage stratified cluster random sampling was adopted to acquire the study sample. The socioeconomic development level among cities was used as stratification standard at the first stage to randomly select three sample areas (Wuhan, Shiyan, and Jingmen, representing high, middle, and low levels of socioeconomic development, respectively) from 13 cities of this province; then, the grade of state-owned medical institutions was used as stratification standard in each city at the second stage to randomly select 67 state-owned medical institutions (8 third-grade institutions, 12 second-grade institutions, and 47 first-grade institutions, representing high, middle, and low referral levels of medical technology and scale of healthcare institutions, respectively); at last, the sample group was selected from these state-owned medical institutions by simple random sampling at the third stage.

This study was approved by the ethics committee of the School of Public Health, Fudan University (IRB#2010090238), and was therefore performed in compliance with the Helsinki Declaration of 1964. All participants in the study were voluntary and expressed informed consent prior to their inclusion.

### Measuring instruments

A five-page questionnaire was produced, consisting of four parts along with a covering letter outlining the survey objective and reply methods.

• Part 1 included the basic sociodemographic information of gender, age, marital status, education background, employment mode, average monthly income (US$), technical position, department, and years of service.

• An assessment of job satisfaction in Part 2 was made using the Chinese Physicians' Job Satisfaction Questionnaire (CPJSQ), with a total of 62 items compiled by Yin WQ et al (2007) [33] and well validated in a subsequent large-scale research [34]. Now the instrument can be only available in the Chinese Journal Full-text Database (CJFD). However, minor deletions, including three items even more attached to the scope of social support network, and another two items about the scientific research and clinical teaching work not suitable for physicians from the first-grade community medical institutions, and some other modifications, increasing an important new item about the reasonableness of performance appraisal system for public hospitals, and classifying and re-arranging these remaining measurement items in accordance with the order of work and occupational factor, organizational factor and social factor, were made in order to further improve the quality of this instrument and their ease of use. At last, the questionnaire through these revisions above consisted of 58 specific questions, which participants were asked to rate on a five-point Likert scale from 1 (strongly disagree) to 5 (strongly agree), according to how they perceived each aspect had contributed to their work satisfaction.

Exploratory factor analysis (EFA) of this questionnaire on the pilot sample showed that five factors were extracted by the method of principal components, including job-itself satisfaction, estimated by 10 items; work environment satisfaction, estimated by 13 items; job rewards satisfaction, estimated by 13 items; organizational management satisfaction, estimated by 11 items; and medical practicing environment satisfaction, estimated by 11 items. Then, confirmatory factor analysis (CFA) of this instrument on the formal sample revealed that a five-factor structure Model F could be ideally supported by the investigation data (GFI, NFI, CFI, IFI > 0.9, RMSEA = 0.075, RMR = 0.024, lambda(x) ≧0.55). Furthermore, another hierarchical confirmatory factor analysis (HCFA) Model F', raised on the basis of Model F with these five first-order latent variables, subjected to a higher second-order latent variable (job satisfaction), also reached the goodness-of-fit standards (RMSEA = 0.086, RMR = 0.025, gamma≧0.64). The comparison of chi-square differences on the fitting effectiveness between these two models (⊿χ^2 ^= 10.163, ⊿df = 5, *P *> 0.05) signified that Model F' could be accepted ultimately in line with the principle of parsimony. It indicated by the cross-sample validation that this survey instrument had good construct validity. The Cronbach α coefficient of internal consistency was 0.829 for the job-itself satisfaction subscale, 0.885 for the work-environment satisfaction subscale, 0.916 for the job-rewards satisfaction subscale, 0.924 for the organizational management satisfaction subscale, 0.798 for the medical practicing environment satisfaction subscale, all of which demonstrated a high level of reliability. The scores of the different job-satisfaction subscales were computed to an average score.

• Burnout in Part 3 was estimated using the Chinese Maslach Burnout Inventory (CMBI), with a total of 15 items developed by Li YX (2005) and verified as valid [35]. Only the Likert scale was changed from 7-point to 5-point (ranging from '1, never' to '5, daily') due to the following two reasons: this can keep consistent with Part 2 and 4 on the scale, which is conductive to analyze their relationships more concisely; it seemed to be very difficult for the respondents in the pilot survey to distinguish the answers of 7-point scale. This inventory suitable for the cultural background of China can also be available in the Chinese Journal Full-text Database (CJFD).

EFA of the CMBI on the pilot sample also divided burnout syndrome into three dimensions: emotional exhaustion, the depletion of emotional resources as a result of excessive psychological and emotional demands; depersonalization, an impersonal cynical attitude towards other people; and reduced personal accomplishment, a decreased sense of accomplishment and competence with regard to one's work with clients. Each of these three dimensions was assessed by five items. Further, CFA of this instrument on the formal sample revealed that a three-factor structure Model Q could be appropriately supported by the investigation data (GFI, NFI, CFI, IFI >0.9, RMSEA = 0.068, RMR = 0.047, lambda(x) ≧0.59). Base on Model Q, a HCFA Model Q' with these three first-order latent variables subjected to a higher second-order latent variable (occupational burnout), reached the same fitting indexes with it (⊿χ^2 ^= 0, ⊿df = 0) because of equivalent models. In terms of the famous three-dimension theory on burnout advocated by Maslach C [28], Model Q' could be accepted to a large extent. It indicated by the cross-sample validation that this survey instrument also had good construct validity. Reliability test showed that the Cronbach α coefficient of internal consistency was 0.802 for motional exhaustion, 0.715 for depersonalization, and 0.864 for reduced personal accomplishment, which were acceptable at least. The average score of each dimension rather than cut-offs was calculated, because the status evaluation of burnout was not the main purpose of this study and more information on the survey data could be retained in the analysis of their roles.

• Turnover intention in Part 4 was measured in reference to the studies by Mobley (1978) and Farh (1998) [9,36], including the following three items with a five-point Likert scale from 1 (highly disagree) to 5 (highly agree): thoughts of leaving; looking for new jobs within one year; and willing to accept other better job chances if available. EFA of this questionnaire on the pilot sample indicated that a single factor, named by turnover intention, was extracted by the method of principal components. Afterwards, CFA of this instrument on the formal sample revealed that an one-factor saturated Model W could be perfectly supported by the investigation data (χ^2 ^= 0, GFI, CFI, NFI = 1), with three standardized factor loadings (lambda(x)) evaluated by 0.75, 0.60, and 0.73. This displayed by the cross-sample validation that the survey instrument also had good construct validity. Additionally, the Cronbach α coefficient reflecting the internal consistency was 0.766 for the turnover intention, which demonstrated a moderate level of reliability. The score of turnover intention was also computed to an average score of these three items.

### Data collection

A cross-sectional survey was carried out in March 2010 in 67 state-owned medical institutions from three urban areas. All investigators underwent training together in advance. Participants who were not informed of the specific research hypothesis were anonymously surveyed with the self-administered questionnaire. Each participant was told about the significance and value of this study for improving their interests, and meanwhile presented a small memento to raise response rate as much as possible. Completed questionnaires were regained under detailed inspections on the spot by the investigators, who had no access to the subsequent material arrangement and database construction. Data was accepted for final analysis after invalid questionnaires (46) were rejected on the basis of the following three principles: sociodemographic data incomplete; more than three missing values among all items; and responses showing undulating curve or over-centralized, the latter two of which may lead to systematic errors in the measurement process and affect the validity of this study.

### Statistical analysis

Data analysis was performed with SPSS for Windows Version 16.0 and AMOS Version 7.0. The threshold of statistical significance was set at *P *< 0.05 (two-tailed) at least. First, on the basis of descriptive statistics (+/-S) on turnover intention, comparisons were made among groups from the three urban areas with different development levels and urban state-owned medical institutions with different grades by one-way ANOVA test and post hoc least-significant difference (LSD). Then, Pearson correlation coefficients were calculated to determine the associations among job satisfaction, burnout syndrome dimensions and turnover intention. One thing which needs to be pointed out is that these numerical variables can be deemed as continuous through averaging corresponding items weighted by the factor loadings of CFA models above, and also meet the normal distribution via hypothesis testing.

Moreover, GLM-univariate analysis was employed, mainly to examine whether job satisfaction and occupational burnout dimensions among physicians could significantly affect turnover intention regarded as a dependent variable. This analysis was also used to assess the changes in significance, parameter size, and explained variance (Adjusted R^2^) by building progressive models, especially when sociodemographic characteristics were controlled as additional independent variables.

**Figure 1:**
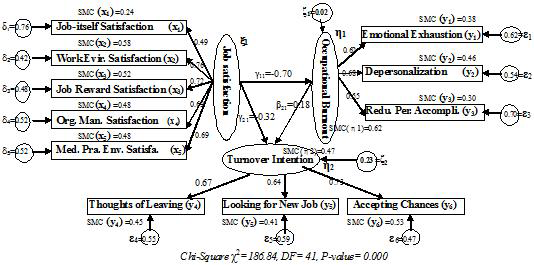
**The path and final solution (R**_**T**_**) of Model R on synthesized relationship between job satisfaction, occupational burnout and turnover intention among physicians**.

Next, structural equation modeling (SEM) with latent variables, in which measurement error could be excluded in the process of parameter estimations, was applied to further verify the hypothetical relationship among the three concepts of job satisfaction, occupational burnout, and turnover intention of physicians in urban state-owned medical institutions in China. The hypothetical Model R was created in light of the attitude theory and some related researches; it is presented in Figure 1. Three measurement models were constructed as follows: job satisfaction (xi_1_) as an exogenous latent variable affecting five exogenous observed variables (x_1-5_), acquired by item parceling with respective measurement errors estimated (delta_1-5_); occupational burnout (eta_1_) as an endogenous latent variable affecting three endogenous observed variables (y_1-3_), also acquired by item parceling with respective measurement errors estimated (epsilon_1-3_); turnover intention (eta_2_) as the ultimate endogenous latent variable, affecting three endogenous observed variables (y_4-6_) with respective measurement errors estimated (epsilon_4-6_). The structural path hypothesis included in Model R is that job satisfaction (xi_1_) has a direct and also indirect effect through occupational burnout (eta_1_), serving as a mediator with an unexplained disturbance (zeta_1_) on turnover intention (eta_2_) with another unexplained disturbance (zeta_2_). Factor loading of any one of the observed variables in every measurement model was designated 1, with the variance of these three latent variables estimated freely. Two-stage modeling rule was adopted to test the identification of Model R [37]. This part of the measurement models met the three indicating criteria: each latent variable affecting not less than three observed variables; each observed variable influenced only by a single latent variable; and no correlation hypothesis between measurement errors. It could therefore be identified effectively. In addition, this part of the structure model was also automatically identifiable since it conformed to the sufficient condition of the recursive criterion, in which the beta matrix (ΒE) was subdiagonal and the psi matrix (PS) diagonal. Maximum likelihood (ML) was utilized to estimate these parameters in Model R.

Finally, the invariance in this assumed model among different samples was tested by means of multi-group SEM to inspect its stability. The fundamental principle was to establish several nested models with restrictions imposed gradually and prove the invariance of restricted parameters by comparing the differences of goodness of fit (⊿χ^2^) between these models based on assuming the previous model to be correct [38].

## Results

### Sociodemographic characteristics of study group

Of the 1600 physicians surveyed in the three urban areas, 1497 (93.6%, response rate) responded. Of those responses, 1451 valid questionnaires were finally acquired, yielding an effectiveness rate of 96.9%. Of the study sample, 501 doctors were from Wuhan city, with 476 from Shiyan city and 474 from Jingmen city; 594 doctors were from the third-grade medical institutions, with 489 from the second-grade medical institutions and 368 from the first-grade medical institutions. The sociodemographic characteristics of study group were shown in Table 1. It was specially seen from it that 440 doctors (30%) received low levels of education (junior college and below), most of which worked in the first and second-grade medical institutions and provided basic medical and public health services within the community in China, and a total of 164 (almost 12%) were medical assistants, the technical titles of which had not usually been identified since these doctors entered their organizations for less than two years after graduation, or they were restricted by the threshold of education background.

**Table 1:** Socio-demographic data of the sample group participated in this study(*n *= 1451)

Characteristics	Category	NO.(%)
**Gender**	Male	960 (66.2)
	Female	491 (33.8)

**Age (years)**	≤ 30	556 (38.3)
	31-40	540 (37.2)
	41-50	240 (16.6)
	≥ 51	115 (7.9)

**Marital status**	Single	324 (22.3)
	Married	1127 (77.7)

**Education Background**	Technical Secondary School	143 (9.9)
	Junior college	297 (20.5)
	Bachelor	787 (54.2)
	Master	164 (11.3)
	Doctor	60 (4.1)

**Years of service**	≤ 5	549 (37.9)
	6-15	604 (41.6)
	16-25	233 (16.0)
	≥ 26	65 (4.5)

**Employment mode**	Formal	1192 (82.2)
	Casual	259 (17.8)

**Technical title**	Medical Assistant	164 (11.3)
	Resident Physician	466 (32.1)
	Attending Physician	477 (32.9)
	Associate chief Physician	270 (18.6)
	Chief physician	74 (5.1)

**Department**	Internal Medicine	412 (28.4)
	Surgery	382 (26.3)
	Pediatrics	49 (3.4)
	Ophthalmology and Otorhinolaryngology	122 (8.4)
	Prevention and Care	32 (2.2)
	Medical laboratory	162 (11.2)
	Gynaecology & Obstetrics	132 (9.1)
	Anesthesiology	110 (7.6)
	Emergency	50 (3.4)

**Average income/month ($)**	≤152	43 (3.0)
	153-304	537 (37.0)
	305-456	429 (29.6)
	457-608	257 (17.7)
	≥609	185 (12.7)

### Statistical description and comparisons of turnover intention

Table 2 shows the observed mean (3.18 +/-0.73) of turnover intention, its score varying from one to five points among the sampled physicians from urban state-owned medical institutions. The results of the ANOVA test indicate that there was a significant difference in turnover intention among these groups from the three urban areas representing different levels of socioeconomic development (F = 9.513, *P *< 0.01); there was likewise no significant difference among these groups with regard to grade of urban state-owned medical institution (F = 0.319, *P *> 0.05). Furthermore, multiple comparisons through post hoc LSD test show that the turnover intention of physicians from Shiyan, with medium-level development, was obviously higher than that for the other two urban areas, with high- or low-level development, between which there was no significant difference.

**Table 2:** Mean and comparisons among groups in turnover intention

		*n*		*S*	F	*P value*
**Socioeconomic development level of urban area**	high level(Wuhan)	501	3.11	0.70	9.513	0.000
	medium level(Shiyan)	476	3.29	0.73		
	low level(Jingmen)	474	3.13	0.74		

**Grade of state-owned medical institution**	first grade	368	3.19	0.71	0.319	0.727
	second grade	489	3.15	0.74		
	third grade	594	3.18	0.72		

**Total sample**		1451	3.18	0.73	--	

### Correlation between job satisfaction, burnout syndrome dimensions, and turnover intention

Table 3 shows that the turnover intention of Chinese physicians is significantly and negatively related to the five subscales of job satisfaction (*P *< 0.01), but positively related to each subscale of burnout syndrome (*P *< 0.01). When the correlation between turnover intention and job satisfaction was examined, it could be seen that there were comparatively higher correlation coefficients between turnover intention and organizational management satisfaction (*r *= -0.323) and work environment satisfaction (*r *= -0.306) than with other dimensions, the correlation coefficients of which were less than 0.30. Among the three dimensions of burnout syndrome, turnover intention showed the highest correlation with emotional exhaustion (*r *= 0.229) and the least with reduced personal accomplishment (*r *= 0.114).

**Table 3:** Correlation between job satisfaction, burnout syndrome dimensions, and turnover intention

		Pearson correlation coefficient with turnover intention	*P *value
**Job satisfaction dimensions**	job-itself satisfaction	-0.203	0.000
	work environment satisfaction	-0.306	0.000
	job rewards satisfaction	-0.293	0.000
	organizational management satisfaction	-0.323	0.000
	medical practicing environment satisfaction	-0.213	0.000

**Burnout syndrome dimensions**	emotional exhaustion	0.229	0.000
	depersonalization	0.211	0.000
	reduced personal accomplishment	0.114	0.000

### Effect of job satisfaction and burnout syndrome dimensions on turnover intention

The data were further subjected to three GLM-univariate analyses with turnover intention as the explained variable, where the job satisfaction and burnout syndrome of physicians were taken as principal predictors with their sociodemographic characteristics simultaneously used as controlled variables.

It is evident in Table 4 that such variables as gender, age, years of service, and average monthly income contributed significantly to turnover intention, with the total of 3.3% criterion variance explained when only the sociodemographic variables were admitted in Model K_1 _(F = 2.692, *P *< 0.01)_. _Univariate analysis in Model K_2 _(F = 8.928, *P *< 0.01) built with job-satisfaction dimensions added on the basis of Model K_1 _showed that a set of independent variables jointly accounted for 29.7% variance of the turnover intention; it also showed that job-itself satisfaction (*b *= -0.060, *P *< 0.01), work environment satisfaction (*b *= -0.075, *P *< 0.01), job rewards satisfaction (*b *= -0.084, *P *< 0.01), and organizational management satisfaction (*b *= -0.142, *P *< 0.01) among job-satisfaction variables proved to be negative predictors of turnover intention under the control of sociodemographic variables, among which the gender, age, and years of service variables were significant. Model K_3 _(F = 9.181, *P *< 0.01) in Table 4, which was established with burnout syndrome dimensions added based on Model K_2_, showed that 41.2% of the variance in turnover intention is covered; it also showed that work environment satisfaction (*b *= -0.074,*P *< 0.01), job rewards satisfaction (*b *= -0.073, *P *< 0.01), and organizational management satisfaction (*b *= -0.146, *P *< 0.01) among job-satisfaction variables, and emotional exhaustion (*b *= 0.135, *P *< 0.01) among burnout syndrome variables, were identified as significant direct predictors of turnover intention under the control of sociodemographic variables, among which these three variables as gender, age, and years of service were still significant.

**Table 4:** Results of univariate analyses by building progressive models with turnover intention as dependent variable

Explanatory Variables		**Model K**_**1**_		**Model K**_**2**_		**Model K**_**3**_	
		
		F	*P *value	F	*P *value	F	*P *value
**Sociodemographic variables※**	gender	8.289	0.004	9.969	0.002	8.861	0.003
	age	5.807	0.001	4.668	0.003	4.105	0.007
	marital status	0.997	0.318	0.321	0.571	0.085	0.771
	education background	0.467	0.760	0.955	0.431	0.996	0.408
	years of service	3.521	0.015	3.982	0.008	3.915	0.008
	technical position	0.157	0.960	0.593	0.668	0.382	0.821
	department	0.954	0.471	0.945	0.478	0.877	0.535
	average monthly income	2.858	0.022	1.963	0.098	2.176	0.069
	employment mode	0.528	0.468	0.867	0.352	1.337	0.248

**Job satisfaction variables (*b*, 95%CI)**	job-itself satisfaction			6.667	0.010	3.679	0.055
				-0.060 (-0.106, -0.015)		-0.053 (-0.107, 0.001)	
	Work environment satisfaction			7.936	0.005	7.822	0.005
				-0.075 (-0.127, -0.023)		-0.074 (-0.125, -0.022)	
	job rewards satisfaction			14.655	0.000	10.633	0.001
				-0.084(-0.128, -0.041)		-0.073 (-0.117, -0.029)	
	organizational management satisfaction			31.593	0.000	34.223	0.000
				-0.142 (-0.191, -0092)		-0.146 (-0.195, -0.097)	
	medical practicing environment satisfaction			0.109	0.742	0.813	0.367
				-0.007 (-0.050, 0.036)		-0.021 (-0.068, 0.026)	

**Burnout syndrome variables (*b*, 95%CI)**	emotional exhaustion					26.756	0.000
						0.135 (0.084, 0.186)	
	depersonalization					0.009	0.924
						0.003 (-0.058, 0.064)	
	reduced personal accomplishment					1.132	0.287
						0.043 (-0.036, 0.121)	

**Corrected Model**		2.692	0.000	8.928	0.000	9.181	0.000
**Adjusted R**^**2**^		0.033		0.297		0.412	

^**※**^the last category in every sociodemographic variable defined as reference.

### Synthesized relationship among job satisfaction, occupational burnout, and turnover intention as congregated latent concepts

Model R finally reached convergence status by nine-times iteration on the data of the total sample. The received standardized regression weights and squared multiple correlations (SMC) of these observed variables and endogenous latent variables were shown in Figure 1. Both five factor-loading parameters (0.49≦lambda(x) ≦0.76) in lambda(x) matrix and another six factor-loading parameters (0.55≦lambda(y) ≦0.73) in lambda(y) matrix met the significant level (*P *< 0.01). In the structural path model, job satisfaction had significant direct effect on occupational burnout (gamma_11 _= -0.70, *P *< 0.01) and turnover intention (gamma_21 _= -0.32*, P *< 0.01); occupational burnout had significant direct effect on turnover intention (beta_21 _= 0.18*, P *< 0.01). Moreover, there still was significant indirect effect (gamma_11 _× beta_21 *= *_-0.13, *P *< 0.01) of job satisfaction on turnover intention through occupational burnout as a mediator. Table 5 shows the integral model-fit effectiveness of Model R_T _by such evaluation indexes as χ^2^**/**df = 4.557, GFI = 0.908, NFI = 0.904, IFI = 0.912, CFI = 0.911, RMR = 0.053, and RMSEA = 0.077, all of which approached the standards of experience value. According to modification indexes (MI), two paths focusing on measurement residuals were recommended for inclusion in this model, involving the covariance between delta_5 _and epsilon_2 _(MI = 15.53), and between delta_1 _and epsilon_3 _(MI = 12.22). However, no modification was executed because they were contrary to the premise of no correlation between delta and epsilon in SEM. So the results of parameter estimation above were accepted as final solution of Model R_T_.

**Table 5:** Goodness-of-fit indexes of Model R on samples from different urban areas

MODEL	χ^2^/df	GFI	AGFI	NFI	IFI	CFI	RMR	RMSEA
**Experience value**	2.0-5.0	>0.90	>0.90	>0.90	>0.90	>0.90	<0.10	<0.08
**Model R_T_(Total sample)**	4.557	0.908	0.897	0.904	0.912	0.911	0.053	0.077
**Model R_W_(Wuhan)**	4.693	0.904	0.893	0.900	0.908	0.909	0.058	0.077
**Model R_S_(Shiyan)**	3.370	0.911	0.902	0.906	0.912	0.912	0.050	0.076
**Model R_J_(Jingmen)**	4.187	0.905	0.893	0.903	0.910	0.911	0.054	0.077

In addition, the goodness of fit of Model R on single samples from the three different urban areas exhibited in Table 5 could also be accepted; there was little difference among these. To test further the invariance of parameters on this model among these three samples, six nested models were established by multi-group SEM in Table 6. Compared with the previous model separately, it was found that there were no significant changes in goodness of fit for the measurement weights invariance model (⊿χ^2 ^= 21.528, ⊿df = 16, *P *> 0.10) and structural weights invariance model (⊿χ^2 ^= 11.207, ⊿df = 6, *P *> 0.05). But, the changes in goodness of fit were significant in the last three restricted models (*P *< 0.01).

**Table 6:** Multigroup invariance test on Model R among three samples from different urban areas

MODEL (Assuming previous model to be correct)	⊿χ^2^	⊿df	*P value*	NFI Delta-1	IFI Delta-2	RFI rho-1	TLI rho2
**Unconstrained model**	--	--	--	--	--	--	--
**Measurement weights invariance model**	21.528	16	0.159	.010	.011	-.013	-.014
**Structural weights invariance model**	11.207	6	0.082	.006	.006	-.005	-.005
**Structural covariance invariance model**	10.439	2	0.005	.002	.002	-.004	-.004
**Structural residuals invariance model**	14.911	4	0.005	.003	.003	-.003	-.003
**Measurement residuals invariance model**	42.585	22	0.005	.007	.007	-.019	-.020

## Discussion

It needs to be pointed out that the working practices and policy-related environment that Chinese physicians usually face are very different between urban areas and rural areas, as well as between public medical institutions and private medical institutions. So our findings are not representative of the situation for physicians in rural areas and private medical institutions in this province. It can be seen from the sociodemographic structure of the study group that the distributions of such variables as age, technical title, and average monthly income form a pyramid shape, which is in accordance with the basic features of this industry. We also have to note these typical characteristics (largely male, mostly fewer than 40 years, and earning less than $456 a month) of our respondents when comparing our results with those of other studies.

This study revealed that the physicians from urban state-owned medical institutions experienced a moderate degree of turnover intention, which was slightly lower than our expectations. We tentatively put forward that this may be related to the new medical reform in a way, began from 2009. The differences with the old one are that the compensation mechanism for hospitals and doctors will be changed from by drug to by services, and the lifelong relationship system between hospitals and doctors will be replaced by the fixed-term engagement system. There is a greater tendency for physicians to leave their practices owing to problems with aspects of their work since previous barriers on the free movement of workers have gradually been reduced. However, it seems to be difficult for physicians to make decisions under the uncertain market conditions created by this system reform, because there is no assurance regarding their future prospects if they leave their present departments.

With regard to differences in turnover intention related to the development level of urban areas, our analysis indicated that the turnover intention of physicians from an urban area of medium-level development was significantly highest. In fact, this can be fully explained by Model K_2 _and K_3_, which displayed that some dimensions of job satisfaction and occupational burnout had predictive effects on the turnover intention. We learned that this group had the evidently greater dissatisfaction with work circumstances and more serious emotional exhaustion, making them more desirous to leave than the other two groups. In particular, salaries out of proportion with local cost were cited most frequently by this group as being a reason behind their intention to go, through the classification of attributions and comparison on their occurrences among different urban areas in the face to face in-depth interviews (40 minutes each person) of some physicians, acquired from these three cities by purposive sampling. In addition, as the grade of state-owned medical institutions rose, the job satisfaction of physicians was on a declining curve and the overall occupational burnout also became more serious, but it was not associated with the turnover intention of physicians. The main reason is that physicians in the high-grade medical institutions are usually unwilling to submit to basic medical units in order to maintain their social reputations and statuses, while those in the community health service centers also have the turnover intention, even if they are satisfied with their present jobs, due to the mentality that man struggles upwards and water flows downwards.

According to the Model K_1_, K_2 _and K_3 _in univariate analysis, gender, age and years of service were three important demographic variables to influence the turnover intention of physicians, which is different from the results on medical personnel by Shao H et al [39]. The latter held that age and gender had no significant effect on their turnover intention. This study indicated that the turnover intention of male physicians was higher than that of females, which is possibly related to men's traditionally strong achievement motivation and their prevailing values of venturing out; meanwhile, as physicians at aged 31-40 with 6-15 years of service had a relatively higher dissatisfaction with working conditions, this group showed the strongest turnover intention based on their pursuing of career success and desire for a change of the present working and life status. Besides, since the variable of average monthly income became no longer significant in Model K_2_, this research deemed that this variable probably acted on the turnover intention of physicians via job satisfaction.

In terms of the correlation between job satisfaction and turnover intention among physicians, all subscales of job satisfaction were found to be negatively related to turnover intention, which is consistent with the studies of Shao H et al and Griffeth R.W et al [39,40]. Pathman DE et al also concluded that turnover intention of clinical physicians was significantly associated with relative dissatisfaction with reward and teamwork [24]. However, another empirical study noted that there were no correlations between social status satisfaction (*r *= -0.041, *P *= 0.32), work conditions satisfaction (*r *= -0.017, *P *= 0.68), and doctor-patient relationship satisfaction (*r *= -0.070, *P *= 0.09) and turnover intention among physicians [25]. Concerning the correlation between burnout and turnover intention, our results confirm that the three dimensions of burnout are positively related to turnover intention, which has been demonstrated in previous studies [31,41,42], whereby physicians suffering from serious burnout tend to report higher degrees of turnover intention. However, a study on nurses revealed that there was correlation only between emotional exhaustion (*r *= 0.321, *P *< 0.01) and turnover intention [43]. From the size of the Pearson correlation coefficients, we tentatively suggest that the association between job satisfaction and turnover intention was relatively greater than that between burnout syndrome and turnover intention.

Under the control of sociodemographic variables, the results of Model K_2 _indicated that the other four satisfaction dimensions (except medical practicing environment satisfaction) had a significant impact on turnover intention. From a comparison between Model K_2 _and K_1_, it is known that the variance with an additional value of 26.4% on turnover intention was explained by job satisfaction exclusively. An influential study undertaken by Chen M et al disclosed that leadership behavior attached to organizational management and teamwork spirit attached to work environment had no predictive effect on turnover intention of company employees [44]. This is probably a consequence of industry discrepancy. However, Yin WQ et al surveyed community doctors and reported that the medical environment satisfaction (*b *= -0.072, *P *< 0.05) and doctor-patient relationship satisfaction (*b *= -0.033, *P *< 0.05) also affected turnover intention [45]. Meanwhile, our results do not completely correspond with a study focusing on third-grade public hospitals by Gu ST et al, which found by use of stepwise linear regression that job-itself (*b *= -0.059, *P *< 0.01), professional safety (*b *= -0.128, *P *< 0.01), and leadership behavior (*b *= -0.043, *P *< 0.01) among thirteen job-satisfaction elements were the best predictive combination of turnover intention among clinical physicians [25]. These inconsistencies might stem from the differences in the specific sample source or real changes of various factors owing to the transition period of medical reform.

After the burnout syndrome variables were included in Model K_3_, only the emotional exhaustion among them was a significant predictor of turnover intention, which was also noted by Li YM et al [43]. This is partially consistent with Huang IC et al [32], who revealed that both exhaustion and cynicism could affect turnover intention in a non-service profession. From a comparison with Model K_2_, it was clear that 11.5% of the variance on turnover intention could be accounted for alone by burnout; job-itself satisfaction no longer became significant, with the estimated parameter on job rewards satisfaction smaller. So, it can be explicitly stated that turnover intention was explained more by job satisfaction than by burnout. Combined with our pre-analysis on the relationship between job satisfaction and burnout, suggesting that such elements as job-itself satisfaction (*b *= -0.166, *P *< 0.01), job rewards satisfaction (*b *= -0.096, *P *< 0.01), and medical practicing environment satisfaction (*b *= -0.084, *P *< 0.01) had predictive effects on emotional exhaustion, we inferred that job-itself satisfaction and job rewards satisfaction would yield indirect effects on turnover intention through emotional exhaustion. This was not found for medical practicing environment satisfaction, since it could not explain significantly turnover intention in Model K_2_. In line with the size of regression parameters in Table 4 and correlation coefficients in Table 3, the results imply that more importance should be attached to organizational management satisfaction, covering two aspects of system construction and leadership behavior, and emotional exhaustion, serving as a mediator for maintaining physicians' stability. Besides, attention has to be paid to job rewards satisfaction, generating both direct and indirect effects on turnover intention.

Further, SEM, known as the integrated model in multivariate statistics, was employed to verify the synthesized relationship among job satisfaction, occupational burnout, and turnover intention, treated entirely as congregated latent concepts for the first time. As the HCFA Model F' and Q' and Model W' could be accepted in the inspection of measuring tools, this provides a reasonable measurement basis for the later assessment of the structural model. Similarly, it also allowed Model F' and Q' with good fittings to be demoted to two first-order CFA models through item parceling, which turned their first-order latent factors into observed variables, so as to simplify these two measurement models during model specification [37]. Our results in Figure 1 indicate that all factor-loading parameters and squared multiple correlations (SMC = lambda^2^) of the observed variables, except job-itself satisfaction, were within the acceptable range (lambda≧0.55) suggested by Tabachnick & Fidell [46], revealing that the measurement errors (1-SMC) of these observed variables accounted for a relatively small proportion; these results also indicate that the direction and size of three structural parameters, which were significant, basically conformed to theoretical expectations. Since the χ^2 ^test trying to support the null hypothesis in SEM evaluation is easily influenced by degree of freedom (df) and sample size, we computed the normed chi-square (χ^2^/df) instead of χ^2 ^to reflect the fitting of model [47]. The goodness-of-fit indexes in Table 5 show that the fitting effectiveness of Model R entirely achieved an acceptable level, suggesting that this model can be supported by the total sample and also by three single samples from different urban areas. Moreover, the test of multigroup invariance on Model R by comparison of nested-model fitting chi-square differences demonstrates that the measurement weights (lambda(x), lambda(y)) and structural weights (gamma, beta) were equivalent among these three different samples, signifying that there was a certain degree of stability in this model. According to Hou JT et al [48], it was extremely difficult to achieve the invariance of structural residuals and measurement residuals in multi-group SEM. Thus, it can be concluded from our study that this hypothetical Model R was appropriate for the observed data, and job satisfaction had a total effect of -0.45 on turnover intention, part (-0.13) of which was generated indirectly through the intermediary role of occupational burnout. Combined with the results of path analysis above among different dimensions, we further prudently inferred that this intermediary effect of occupational burnout could mainly be from the dimension of emotional exhaustion among it.

In light of the estimated SMC scores of two endogenous latent variables, it is known that 62% of the variance in occupational burnout (eta_1_) and 47% of the variance in turnover intention (eta_2_) are explained by this SEM. Compared with Adjusted R^2 ^in Table 4, we found that more variance (9%) in turnover intention could be accounted for by both job satisfaction and burnout when the measurement errors of these observed variables were excluded. However, we could not be completely certain that this assumed Model R was absolutely true of the theoretical relationship among concepts, even if it had been verified as being in line with the observed data by obtaining ideal fittings; this is because there may also be other supported models constructed by the same variables [37,48,49]. So, it is important to avoid the trap of over-inference. In addition, it has to be acknowledged that part of the evaluation effectiveness on Model R originated from item parceling of HCFA Model F' and Q', which decreased the number of elements in the observed covariance matrix and narrowed the residuals between the reproduced covariance matrix and observed covariance matrix in the process of model fitting [50].

The main limitation in this study was a cross-sectional design, which often inferred causal effect with low power, as well as an inherent defect in SEM, as discussed above. Additional research, in which organizational commitment is also included, is necessary to confirm these relationships by comparing competitive models or longitudinal design. The second limitation was that such measurement tools as job satisfaction and occupational burnout we used, which were obtained by modifying the CPJSQ and CMBI, respectively, were non-standard instruments. Third, the extrapolation of conclusions would be limited to some extent, as the participants were chosen only from central China's Hubei Province, owing to some constraints of time and convenience; however, we are still encouraged by the fact that the demographics of the study sample were similar to those of a corresponding group reported in the Fourth National Health Services Survey by the Ministry of Health of China. The last limitation was that the sample size was relatively small if we take into account the total number of this population, and these tests of association about ANOVA, Univariate analysis and SEM were potentially underpowered as the sample size calculation was based on an estimate of a population mean.

## Conclusions

Our study, underlining the importance of the psychological attitude at work, indicates that several, but not all dimensions of both job satisfaction and burnout syndrome prove to be relevant factors affecting physicians' turnover intention. In particular, it suggests that there may be partial mediation effects of occupational burnout, mainly through emotional exhaustion, within the impact of job satisfaction on turnover intention, among Chinese physicians in urban state-owned medical institutions, who experience a moderate degree of turnover intention with significant differences according to location. This signifies that enhancements in job satisfaction can be expected to reduce physicians' intentions to quit by the intermediary role of burnout as well as the direct path. It is hoped that these findings will offer some clues for health-sector managers to keep their physician resource motivated and stable.

## List of abbreviations

GFI: goodness-of-fit index; NFI: normed fit index; CFI: comparative fit index; IFI: incremental fit index; RMSEA: root mean square error of approximation; RMR: root mean square residual

## Competing interests

The authors declare that they have no competing interests.

## Authors' contributions

YZ that conceived of the study and carried out its overall design performed the acquisition, analysis and interpretation of data and drafted the manuscript. XF who also participated in the design of this study was involved in revising it critically. All authors approved the final manuscript to be published.

## Pre-publication history

The pre-publication history for this paper can be accessed here:

http://www.biomedcentral.com/1472-6963/11/235/prepub
